# THE NUTRITIONAL STATUS OF HOSPITALIZED CHILDREN AND ADOLESCENTS: A COMPARISON BETWEEN TWO NUTRITIONAL ASSESSMENT TOOLS WITH ANTHROPOMETRIC PARAMETERS

**DOI:** 10.1590/1984-0462/;2017;35;3;00006

**Published:** 2017-07-31

**Authors:** Thaynara Cristina de Oliveira, Izabela Zibetti de Albuquerque, Maria Luiza Ferreira Stringhini, Andrea Sugai Mortoza, Bruna Alves de Morais

**Affiliations:** a Universidade Federal de Goiás, Goiânia, GO, Brasil.

**Keywords:** Child, Adolescent, Anthropometry, Nutritional assessment, Malnutrition, Nutritional status

## Abstract

**Objective:**

Verify the association between anthropometric indicators and the Subjective Global Assessment of Nutritional Status (SGA) and the Screening of Risk for Nutritional Status and Growth (STRONGkids) scales.

**Methods:**

A cross-sectional study with patients from 0 to 18 years admitted in the Hospital das Clínicas, Goiânia (GO), between August and November 2015. Children and adolescents admitted in up to 48 hours were included. Patients who required specific instruments for assessing their nutritional status and those hospitalized in Intensive Care were excluded. Identification and anthropometric data was collected and applied to the SGA and STRONGkids. We performed an analysis comparing proportions and did an agreement assessment, where *p*<0.05 was significant.

**Results:**

71 patients were evaluated, of whom 9.6% had low or very low birth weight/age, 9.7% had thinness or accentuated thinness according to the weight/height index, 16.9% had a height impairment, 7% were thin according to the body mass index/age, and 32.4% were malnourished with regard to arm muscle circumference. The STRONGkids detected that 69% of the sample had a moderate or high nutritional risk. According to the SGA, malnutrition prevalence was 38.1%. There was an association between the SGA and body mass index/age (*p*=0.022), height/age (*p*<0.001) and arm muscle circumference (*p*=0.014). There was no association between the STRONGkids and anthropometric indicators. A correlation was found between: high nutritional risk *versus* severe malnutrition and low nutritional risk x the well-nourished (*p*<0.001), but the agreement was weak (k=0.255).

**Conclusions:**

It is recommended to use the STRONGkids as a screening instrument because it has a higher sensitivity for diagnosing patients with a nutritional risk. The SGA should be applied to nutritional assessment due to its association with anthropometry.

## RESUMO

**Objetivo:**

Verificar associação entre indicadores antropométricos e as escalas Avaliação Nutricional Subjetiva Global (ANSG) e Triagem de Risco para Estado Nutricional e Crescimento (STRONGkids).

**Métodos:**

Estudo transversal com pacientes de 0 a 18 anos, internados no Hospital das Clínicas, Goiânia (GO), entre agosto e novembro de 2015. Foram incluídas crianças e adolescentes admitidos em até 48 horas. Excluíram-se pacientes que requeriam instrumentos específicos para avaliar o estado nutricional e os internados em Terapia Intensiva. Coletaram-se dados de identificação, antropométricos e foram aplicadas a ANSG e a STRONGkids. Feita análise de comparação de proporções e avaliação de concordância, sendo significante *p*<0,05.

**Resultados:**

Avaliaram-se 71 pacientes, dos quais 9,6% com baixo ou muito baixo peso/idade, 9,7% com magreza ou magreza acentuada pelo índice peso/estatura, 16,9% com comprometimento da estatura, 7% com magreza pelo índice de massa corporal/idade e 32,4% desnutridos pela circunferência muscular do braço. A STRONGkids detectou 69% da amostra com risco nutricional moderado ou alto. Pela ANSG, a prevalência de desnutrição foi de 38,1%. Houve associação entre ANSG e índice de massa corporal/idade (*p*=0,022), estatura/idade (*p*<0,001) e circunferência muscular do braço (*p*=0,014). Não houve associação entre a STRONGkids e os indicadores antropométricos. As ferramentas se associaram para: risco nutricional elevado *versus* desnutrição grave e baixo risco nutricional x bem nutridos (*p*<0,001), porém a concordância foi fraca (k=0,255).

**Conclusões:**

Recomenda-se utilizar o STRONGkids como instrumento de triagem por apresentar maior sensibilidade para diagnosticar pacientes com risco nutricional. A ANSG deve ser aplicada para avaliação nutricional devido à associação com a antropometria.

## INTRODUCTION

The global prevalence of primary malnutrition has decreased in recent decades. However, it is unclear whether the prevalence of secondary malnutrition has also decreased.^1^ Children with complex diseases admitted at children’s hospitals share different mechanisms of secondary malnutrition, which are determined by the underlying disease, such as the reduction of food intake, poor absorption and increased energy expenditure, among others. It is possible to assume that the prevalence of secondary malnutrition has no parallel with the trend of primary malnutrition because it is intrinsically linked to different types of morbidities.^1^ ^,^ ^2^

Malnutrition is associated with a bad prognosis in hospitalized patients, and it is possible to identify increased risk of infections, increased loss of muscle mass, difficulty in wound healing, longer hospital stays and increased morbidity and mortality. In children, some additional consequences are added, such as growth and cognitive development impairment as well as low performance at school.^3^ ^,^ ^4^ ^,^ ^5^ In this context, it is important to identify children with the greatest nutritional risk early on, as it enables physicians/clinicians to provide guidelines for an intervention capable of preventing the worsening of the patient’s nutritional status, or for promoting their recovery.^2^ ^,^ ^6^ ^,^ ^7^ ^,^ ^8^

Although anthropometric and body composition data are strong predictors of nutritional risk and they are often used as a single criterion in diagnosing the patient, this data alone does not provide a complete approach. Additional information, such as food intake, clinical status and physical examination, among others, makes the diagnosis different.^4^ ^,^ ^7^ In recent years, several screening and nutritional assessment tools were developed to identify nutritional risk at an early stage. Currently, there are six tools for hospitalized children and adolescents, however, there is no consensus on which is the best tool to use.^8^ ^,^ ^9^ ^,^ ^10^ In clinical practice, the Screening of Risk for Nutritional Status and Growth (STRONGkids) and the Subjective Global Assessment of Nutritional Status (SGA) have been widely used.^8^ ^,^ ^10^

The STRONGkids is considered to be a fast and practical nutritional screening tool, which consists of the analysis of four items: presence of disease with high risk of malnutrition; subjective clinical evaluation; food intake and presence of vomiting or diarrhea; and recent weight loss. It is not necessary to perform anthropometric measurements and, depending on the score obtained, children are classified into high, moderate or low risk of malnutrition.^11^ On the other hand, the SGA demands more time for its application, since it is a more complete and detailed questionnaire used to evaluate and classify the patient’s nutritional status, and addresses anthropometric measurements, physical examination, food intake, gastrointestinal symptoms, functional impairment and metabolic stress of the disease. The child’s nutritional status is assigned to a global ranking of eutrophy/well nourished, moderately malnourished or severely malnourished.^12^ ^,^ ^13^

Thus, the objective of this study is to evaluate the association of the classification of nutritional status obtained by anthropometric indicators, and lean mass with the tools for screening and nutritional assessment, the STRONGkids and the SGA, in pediatric patients and hospitalized adolescents.

## METHOD

A cross-sectional study was conducted with children and adolescents admitted in the pediatric emergency room or in the pediatric ward at the Hospital das Clínicas of Universidade Federal de Goiás (HC/UFG), in the period from August to November 2015. The sampling was performed for convenience, having as inclusion criteria children and adolescents (aged 1 month to 17 years), of both genders, who have been admitted to the hospital in up to 48h (time for the application of the questionnaires). We excluded patients that required specific instruments to assess their nutritional status and those that were admitted to other wards and the Intensive Care Unit (ICU). The way of feeding (oral, enteral and/or parenteral nutrition) was not an exclusion criterion. The parents who agreed to participate in the study signed an Informed Consent Form, and patients aged over six years signed an Informed Assent Form. The project was approved by the Ethics Committee in Human and Animal Research of the Universidade Federal de Goiás in Goiânia (GO), according to Resolution no. 466/2012.

The collection of data occurred in clinics in up to 48 hours after hospital admission, by researchers trained in the application of questionnaires and in the execution of anthropometry. Initially, identification data was collected, such as full name, sex, date of birth, mother’s name and diagnosis of the patient’s records. Subsequently, the STRONGkids and SGA tests were applied, according to anthropometric measurements.

The determination of nutritional status by the STRONGkids varies according to the score obtained in the questionnaire, so that patients are classified as high (4 to-5 points), moderate (1-3 points) and low-risk (0 points). The SGA is a tool that was recently validated for use in the Brazilian population^14^ and its classification system is not numeric, but rather based on critical judgment during the filling out of the questionnaire. For this reason, nutritional status classification as healthy weight/well nourished, moderately malnourished or severely malnourished was acquired by the blind analysis of two researchers, and subsequently the data was cross-checked. The patients who demonstrated a divergence in classification passed through a new analysis to obtain consensus among the researchers.

In the anthropometric assessment, we measured: weight, height, arm circumference and triceps skinfold thickness, with the calculation of body mass index (BMI) and arm muscle circumference. Patients were evaluated according to the following anthropometric indicators: weight for age (0-10 years), weight for height W/H (0-5 years), height-for-age H/A (0-19 years) and body mass index for age, BMI/A (0-19 years), all classified in Z score by the growth curves of the World Health Organization (WHO) in 2006/07,^15^ with the software WHO Anthro version 3.2.2 and WHO Anthro Plus.^16^ For an analysis of arm muscle circumference (AMC), the cutoff points of 11 cm^17^ and 11.5 cm^18^ were used to determine malnutrition in children under the age of 6 months and between 6 and 12 months respectively. For children over 1 year of age, we applied the reference values proposed by Frisancho.^19^

Initially, we used descriptive statistics procedures (frequencies relative and absolute) for categorical variables. To compare proportions, Pearson’s chi-square test was adopted, with *p*<0.05 being significant. In the case of statistical significance, the Adjusted Residual Test was used, considering the absolute value as greater than 1.96 to check local association between categorical variables. To assess agreement between the nutritional screening tools, we applied the Kappa coefficient, and interpreted it according to the value scores proposed by Landis and Koch.^20^ The analyzes were obtained by means of the application SPSS statistical package (IBM SPSS Statistics version 19.0; Chicago, IL, USA).

## RESULTS

A total of 71 patients were included in the study, of which 50.7% were males and 46.5% had some chronic disease (Table 1). The≈median age was 5 years and 2 months. Renal system diseases were prevalent, being present in 25.4% of the patients, followed by cardiorespiratory diseases (15.5%), hematological diseases (9.9%), gastrointestinal diseases (8.5%), rheumatic (7%) and metabolic diseases (5.6%). The other patients (28.2%) had no conclusive diagnosis or did not fit in other disease groups. The patients had an appropriate mean weight and length at birth of: 3220±559 g (n=65) and 49.1±3.1 cm (n=61), respectively.

**Table 1:**
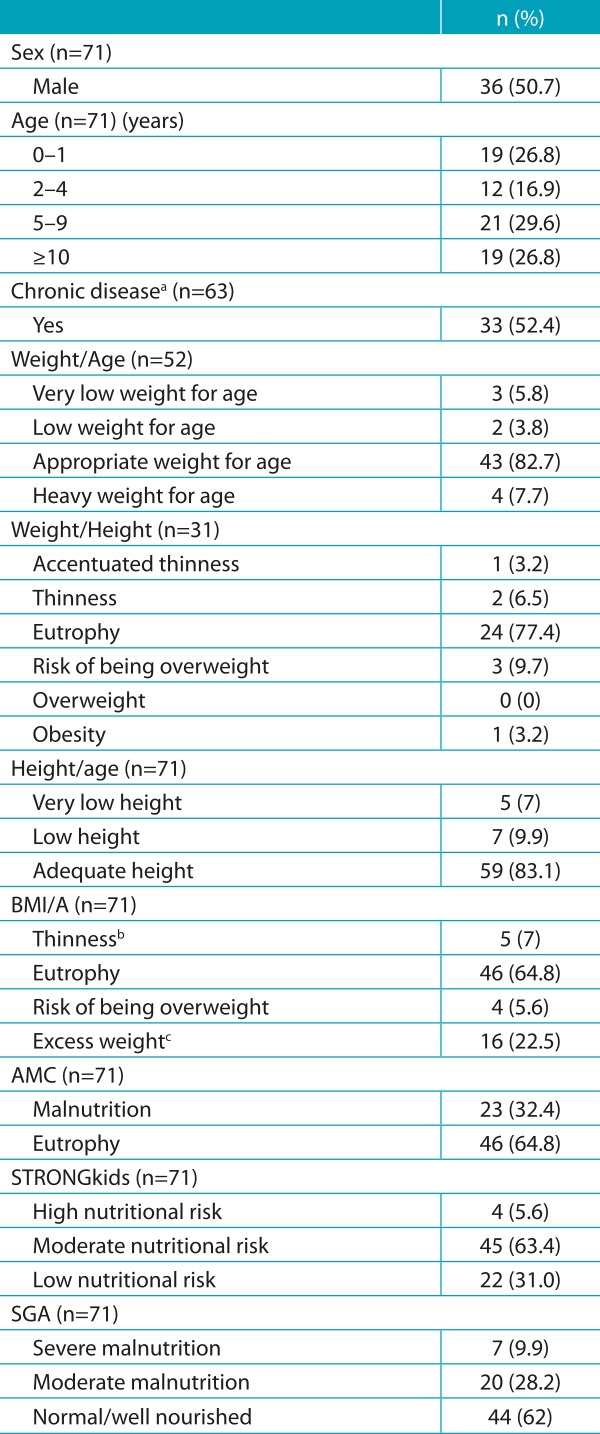
Social and clinical characteristics, and nutritional status of children and adolescents (n=71).

BMI/A: Body mass index for age; AMC: arm muscle circumference; STRONGkids: Screening of Risk for Nutritional Status and Growth; SGA: Subjective Global Assessment of Nutritional Status. ^a^Eight patients had no complete diagnosis; ^b^the category “thinness” included the categories *thinness* and *accentuated thinness*; ^c^the category “overweight” included the categories of *overweight*, *obesity* and *severe obesity*.

When assessed by anthropometric indicators: 9.6% had low or very low birth weight/age; 9.7% thinness or thinness accentuated by weight/height index; 16.9% had a height impairment; 7% were underweight by BMI/A; and 32.4% were malnourished by AMC (Table 1). Through STRONGkids, moderate and high nutritional risk was diagnosed in 69% of the sample. Through the SGA, malnutrition prevalence was 38.1% (Table 1). By correlating the STRONGkids with anthropometric indicators, there was no statistical significance for any parameter analyzed (Table 2).

**Table 2:**
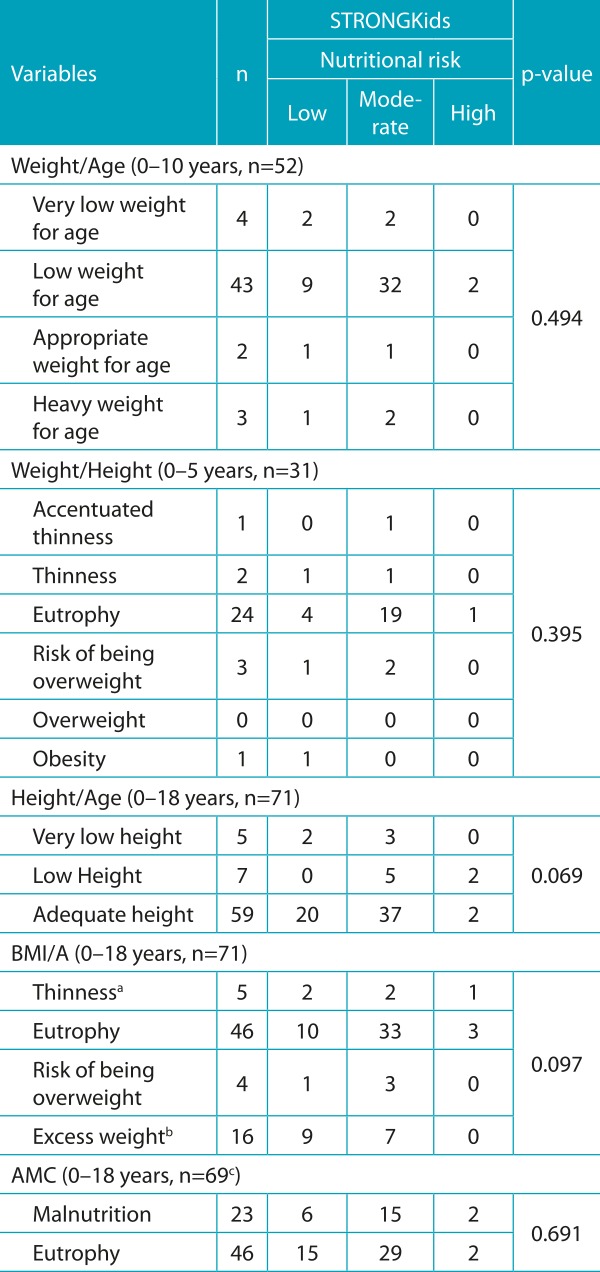
Association between the STRONGkids and anthropometric parameters of children and adolescents.

STRONGkids: Screening of Risk for Nutritional Status and Growth; BMI/I: Body mass index for age; AMC: arm muscle circumference. ^a^The category “thinness” included the categories thinness and accentuated thinness; ^b^the category “overweight” included the categories of overweight, obesity and severe obesity; ^c^two patients did not measure the AMC due to logistical problems.

As for the SGA, it showed a significant association with H/A (*p*<0.001), BMI/A (*p*=0.022), and AMC (*p*=0.014) (Table 3). Through the Adjusted Residual Test, local associations were found between SGA with BMI, AMC and height: low weight *versus* severe malnutrition, overweight *versus* well-nourished, low and very low height *versus* severe malnutrition, adequate height *versus* well-nourished, AMC of malnutrition *versus* moderate malnutrition and AMC suited *versus* well-nourished.

**Table 3:**
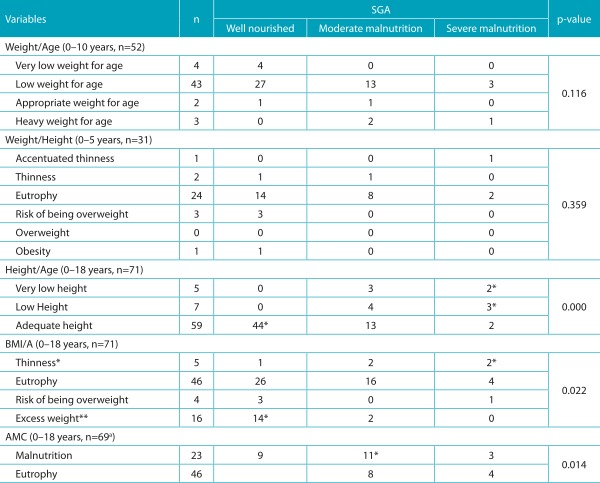
Association between the SGA and anthropometric parameters of children and adolescents.

SGA: Subjective Global Assessment of Nutritional Status; BMI/I: Body mass index for age; AMC: arm muscle circumference. *The category “thinness” included the categories *thinness and accentuated thinness.* *>1.96 by the Adjusted Residual Test; **the category “overweight” included the categories of *overweight*, *obesity* and *severe obesity*. ^a^Two patients did not measure AMC due to logistical problems.

The local association between tools was evident in the following topics: high nutritional risk *versus* severe malnutrition, and low nutritional risk *versus* well-nourished (*p*<0.001) (Table 4). However, through the Kappa Coefficient, the correlation between the two tools was found to be weak (k=0.255).

**Table 4:**
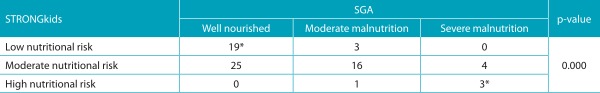
Association between the SGA and STRONGkids of children and adolescents.

SGA: Subjective Global Assessment of Nutritional Status; STRONGkids: Screening of Risk for Nutritional Status and Growth. *>1.96 by the Adjusted Residual Test.

## DISCUSSION

In our study, the STRONGkids showed no association with the anthropometric parameter analyzed. The SGA was associated with BMI/A, H/A and AMC, but not with the W/H and W/A. There was an association between the two tools, although it was of low intensity. It should be emphasized that the STRONGkids is a screening tool with the objective of identifying and categorizing nutritional risk, while SGA aims to evaluate and classify nutritional status.

Corroborating our findings, other studies have demonstrated that, at the time of hospital admission, there is a predominance of children and adolescents with adequate nutritional status when assessed by anthropometric parameters.^3^ ^,^ ^21^ ^,^ ^22^ ^,^ ^23^ On the other hand, weight loss is frequent during hospitalization, which reinforces the importance of early identification of children at risk of deteriorating nutritional status. A prospective study showed that 65% of the children had weight loss during hospitalization and the factors most predictive of its occurrence were: reduced food intake, pain and severity of the disease.^24^

The nutritional screening by the SGA also found a higher prevalence of well-nourished patients than poorly nourished patients in our sample. A study performed in a pediatric hospital in Porto Alegre found that 84.2% of the population was normal/well nourished.^21^ It should be noted that the focus of this tool is to detect malnutrition and, thus, there is a classification for patients with excess weight, which may overestimate the normal category/well nourished. In contrast, 70% of children in a study conducted in Iran showed some degree of malnutrition through the SGA, despite having adequate nutritional status when assessed by H/A and W/A.^23^

This nutritional profile changes significantly when you use STRONGkids. This finding is justified by the presence of a single score that indicates moderate nutritional risk, making the tool quite sensitive.^21^ Moreover, even if the patient does not follow any criteria of the questionnaire, the condition of being hospitalized already considers that he or she is at low nutritional risk. A study carried out in Pelotas also observed a high prevalence of nutritional risk in patients evaluated by the STRONGkids, with 55.3% of the sample having moderate risk and 16% having high risk.^22^ A similar frequency was found in Dutch and Italian hospitals, with 62 and 68% of the patients presenting moderate or high nutritional risk, respectively.^3^ ^,^ ^8^ When the nutritional profile is evaluated according to the type of hospital, a multicenter study carried out in the Netherlands noted a higher prevalence of high nutritional risk in university hospitals, when compared to other hospitals, of 15 and 5%, respectively. The researchers suggest that this is due to a higher number of patients being hospitalized with some chronic disease in university hospitals, in comparison to others.^8^

Also with respect to the STRONGkids, there was no significant association between this tool and anthropometric data. This finding can be explained by the greater sensitivity of the tool in diagnosing nutritional risk. A prospective observational study with 46 children with inflammatory bowel disease also found no association between STRONGkids and diagnosed malnutrition based on anthropometric data proposed by the WHO.^25^ This data was different from that found by Luciana et≈al., who demonstrated a statistical association, although weak, between STRONGkids and BMI/A categories excess weight *versus* low nutritional risk, normal weight *versus* moderate nutritional risk, and malnutrition *versus* high nutritional risk.^21^ A significant, although weak, correlation was also found, between the STRONGkids with BMI/A and H/A in children and adolescents. In the latter, significant correlations between these anthropometric measurements and the STRONGkids were noticed only for the high-risk group.^3^ This data indicates that the tool does not show good correlation with anthropometric indicators commonly used in clinical practice, and that, when this association occurs, it is weak. Such a divergence between the results can be attributed to the fact that the tool does not contemplate anthropometric data in its research. In spite of dealing with two items closely related to anthropometric measurements, such as poor nutritional status verified by physical examination, evaluated by a professional, and the occurrence of weight loss, judged by parents, this data is affected by the subjective analysis imposed. Spagnuolo et al. suggest the consideration of STRONGkids in conjunction with other nutritional parameters due to their numerical system of classification. During this Italian research, many pediatricians pointed out the incompatibility between the clinical judgment of nutritional risk of the patient with the categorization (low, moderate or high risk) produced by the tool.^3^

In relation to SGA, an association of this tool with anthropometric parameters has been reported in the literature, and can be observed in our study for the categories low weight *versus* severely malnourished, excess weight *versus* well-nourished, low and very low height *versus* severely malnourished, adequate height *versus* well-nourished, AMC of malnutrition *versus* moderately malnourished and adequate AMC *versus* well-nourished. In accordance with this result, Campos et al. found a significant association between malnutrition classified by BMI/A and the group of patients with moderate and severe malnutrition by SGA.^21^ When this association was evaluated in critically ill patients admitted in an intensive care unit, a moderate to strong correlation between the weight, height, weight/height, tricipital skinfold, appropriateness of ideal weight and arm muscle circumference with the scores of SGA was found. These findings suggest that SGA can be used in place of anthropometry in critically ill children, as it is the first subjective measure validated to assess the nutritional status of these patients.^26^ Although an association between SGA with four objective parameters of nutritional status (weight, height, triceps skinfold thickness and level of serum transferrin) has not been found, Mahdavi et al. suggest that SGA will be able to identify the risk of malnutrition even before a change occurs in the anthropometric parameters and laboratory tests.^23^ The low correlation found in some studies between SGA and the objective parameters can be justified by some limitations of the tool itself. First, SGA was developed to improve the specificity at the expense of sensitivity; second, it does not classify the patient into mild malnutrition, only into moderate or severe; third, the tool prioritizes chronic nutritional problems, making it difficult to detect acute changes. Despite these limitations and the absence of a numerical system for the final classification of nutritional status, the subjectivity of this tool allows the professional to use clinical judgment rather than apply strict criteria, which may not be valid in the health/disease context.^23^

Although, in some cases, the prevalence of malnutrition has been divergent according to the used tool, an association was found between the SGA and STRONGkids in patients at high nutritional risk and severely malnourished, and at low nutritional risk and well nourished. Although there is no gold standard test, a study evaluated the validity of three screening tools with the SGA, and it was considered to be the most complete method in this study. The STRONGkids showed a sensitivity of 100%, which does not imply a false negative; however, its specificity was of 7.7%, resulting in a false positive of 92.3%.^27^ Recent meta-analysis assessed the accuracy of five nutritional screening tools, including the STRONGkids, for hospitalized children and adolescents, and found no evidence for the selection of a single tool as the most accurate in clinical practice. We suggest, therefore, the use of multiple criteria to select the instrument to be used, such as reliability between evaluators, ease of use and time needed to use the tool.^28^ It is worth noting that the use of screening tools has been recommended by international associations, such as the *British Association of Parenteral and Enteral Nutrition* and the *European Society for Paediatric Gastroenterology, Hepatology and Nutrition*.^25^ ^.^ ^27^ The assessment of nutritional status today only identifies patients who already have some degree of malnutrition, while the early identification of risk of developing it could promote useful nutritional interventions, avoiding the short and long term consequences of malnutrition.^3^

There is no single parameter to define malnutrition in pediatrics; consequently, the assessment of nutritional status becomes quite complex and requires an analysis of various criteria, such as clinical and food history, physical examination, anthropometric and laboratory parameters.^3^ ^,^ ^10^ ^,^ ^13^ Among anthropometric data, it is worth mentioning that the AMC was the most sensitive method to diagnose the impairment of nutritional status, with 32.4% of malnourished patients *versus* 7% with thinness, when evaluated by AMC and BMI/A, respectively. This parameter is associated with total body mass and indicates the association of malnutrition with the reduction of muscle mass. In addition, evidence shows that this depletion is also present in the low height.^29^ Nevertheless, there is little use of AMC in clinical practice.

The major limitation of this study was the fact that many variables addressed in the screening tools and nutritional assessment depend on the memory and/or the judgment of the parents, which may have affected the nutritional diagnosis of the patient. Other possible limitations were:

1. Some patients were discharged before 48 hours of admission, and did not have time for the application to be applie.
2. Sample heterogeneity.
3. Lack of correlation between nutritional status and clinical outcome.
4. The majority of patients did not have the growth curve filled out in the child’s handbook, compromising the reliability of the data.

However, we believe that the research was able to reinforce the need for early assessment as a fundamental step to establish adequate nutritional support for patients.

Although the STRONGkids has not presented an association with anthropometric data, its use is recommended solely as a screening instrument because it presents higher sensitivity for diagnosing patients at nutritional risk. For the assessment of nutritional status, we suggest the use of the SGA, because its results were associated with anthropometry data and allowed an overall analysis of the patient. Thus, we propose that a patient that has moderate or high nutritional risk should be also assessed by the SGA for a nutritional diagnosis and to establish treatment.
